# Crystal Structure of African Swine Fever Virus dUTPase Reveals a Potential Drug Target

**DOI:** 10.1128/mBio.02483-19

**Published:** 2019-10-29

**Authors:** Changyao Li, Yan Chai, Hao Song, Changjiang Weng, Jianxun Qi, Yeping Sun, George F. Gao

**Affiliations:** a College of Veterinary Medicine, China Agricultural University, Beijing, China; b CAS Key Laboratory of Pathogenic Microbiology and Immunology, Institute of Microbiology, Chinese Academy of Sciences, Beijing, China; c Research Network of Immunity and Health (RNIH), Beijing Institutes of Life Science, Chinese Academy of Sciences, Beijing, China; d State Key Laboratory of Veterinary Biotechnology, Harbin Veterinary Research Institute, Chinese Academy of Agricultural Sciences, Harbin, China; e Savaid Medical School, University of Chinese Academy of Sciences, Beijing, China; f National Institute for Viral Disease Control and Prevention, Chinese Center for Disease Control and Prevention (China CDC), Beijing, China; Virginia Polytechnic Institute and State University

**Keywords:** African swine fever virus, dUTPase, E165R, crystal structure, drug target

## Abstract

E165R, a highly specific dUTP nucleotidohydrolase (dUTPase) encoded by the African swine fever virus (ASFV) genome, is required for productive replication of ASFV in swine macrophages. Here, we solved the high-resolution crystal structures of E165R in its apo state and in complex with its product dUMP. Structural analysis explicitly defined the architecture of the active site of the enzyme as well as the interaction between the active site and the dUMP ligand. By comparing the ASFV E165R structure with dUTPase structures from other species, we found that the active site of E165R is highly similar to those of dUTPases from Mycobacterium tuberculosis and Plasmodium falciparum, against which small-molecule chemicals have been developed, which could be the potential drug or lead compound candidates for ASFV. Our results provide important basis for anti-ASFV drug design by targeting E165R.

## Abstract (precis)

African swine fever virus (ASFV), an *Asfivirus* affecting pigs and wild boars with up to 100% case fatality rate, is currently rampaging throughout China and some other countries in Asia. There is an urgent need to develop therapeutic and preventive reagents against the virus. Our crystallographic and biochemical studies reveal that ASFV E165R is a member of trimeric dUTP nucleotidohydrolase (dUTPase) family that catalyzes the hydrolysis of dUTP into dUMP. Our apo-E165R and E165R-dUMP structures reveal the constitutive residues and the configuration of the active center of this enzyme in rich detail and give evidence that the active center of E165R is very similar to that of dUTPases from Plasmodium falciparum and Mycobacterium tuberculosis, which have already been used as targets for designing drugs. Therefore, our high-resolution structures of E165R provide useful structural information for chemotherapeutic drug design.

## INTRODUCTION

African swine fever virus (ASFV), the causative agent of African swine fever (ASF), is a large enveloped double-stranded DNA virus that belongs to the *Asfarviridae* family (1). It is highly contagious and causes lethal hemorrhagic fever in swine, with a mortality rate up to 100% (2). After the first recognition of the disease in Kenya in the 1920s (3), it has spread to many regions of the world, including Europe, South America, the Caribbean, Caucasus, and Asia, causing a devastating blow to the pork industry in the affected regions (4). In mid-June 2018, ASF was first detected in a farm near Shenyang City in Liaoning Province in China (5). Up until 5 September 2019, 156 ASF outbreaks were detected in 32 provinces/autonomous regions/municipalities. Over 1,170,000 pigs have been culled in order to halt further transmission (6). There is no effective vaccine or drug for this disease, and it is urgent to develop preventive and therapeutic reagents against it.

ASFV encodes a dUTP nucleotidohydrolase (dUTPase) (dUTP pyrophosphatase [Dut]; EC 3.6.1.23), called E165R. The enzyme degrades dUTP in the cytoplasm and thus minimizes misincorporation of uracil into viral DNA, which plays an essential role in maintaining the fidelity of genome replication. It is indispensable for reproductive infection of ASFV in swine macrophages, the natural host cell for the virus (7). Therefore, inhibition of the dUTPase activity can be detrimental to ASFV replication and an effective measure of treating ASFV infection.

dUTPases are assumed to ubiquitously exist in nearly all free-living organisms and many DNA and RNA viruses (8). The hydrolysis of dUTP to dUMP and pyrophosphate maintains a low ratio of dUTP/dTTP, which is important to safeguard the DNA-coding information, and simultaneously provides the substrate for thymidine nucleotide biosynthesis (9). These enzymes have several different oligomeric forms, including monomeric (in herpesviruses), homodimeric (in trypanosomatids, some bacteria species such as Campylobacter jejuni, and some bacteriophages), and homotrimeric enzymes (in most eukaryotes, prokaryotes, and some DNA viruses) (10). Crystal structures of dUTPases from many organisms have been determined, including those from *Epstein-Barr virus* (EBV) (11), Leishmania major (12), C. jejuni (13), Trypanosoma cruzi (14), *Feline immunodeficiency virus* (FIV) (15), *Vaccinia virus* (16), Coxiella burnetii (17), Escherichia coli (18), Arabidopsis thaliana (19), Bacillus halodurans (19), Mycobacterium tuberculosis (20), Plasmodium falciparum (21), human (22), among others. Considering their importance in maintaining DNA fidelity, dUTPases from some pathogenic microbes have been used as targets for chemotherapeutic drug design (21, 23, 24).

Here, we present the crystal structures of apo E165R and its complex with dUMP. We show that E165R is a homotrimer and can specifically hydrolyze dUTP. The overall structure and active center of the enzyme are very similar to those of other homotrimeric dUTPases, especially from M. tuberculosis. Our results provide important clues for structure-based drug design by targeting E165R.

## RESULTS

### Characterization of recombinant ASFV E165R protein.

**FIG 1:**
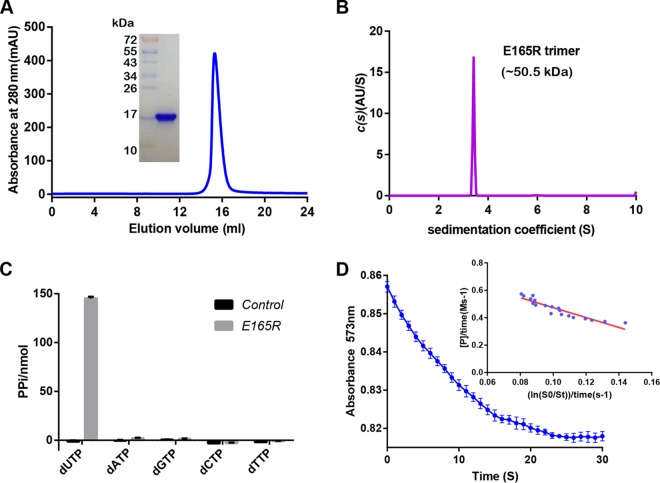
Biochemical characterization of ASFV E165R. (A) Analytical gel filtration of E165R protein. The 280-nm absorbance curve from Superdex 200 10/300 GL column and the SDS-PAGE migration profile of the pooled sample are shown. (B) Ultracentrifugation sedimentation profile of E165R. The calculated molecular weight of the indicated protein species is shown. (C) Substrate specificity assay of E165R showing its specificity for the hydrolysis of dUTP. (D) Enzymatic activity assay of E165R. The *K_m_* values were calculated using the integrated Michaelis-Menten method (42).

Recombinant E165R protein was expressed in E. coli cells as tagged protein with a C-terminal His tag. The Ni-affinity-purified E165R protein was eluted from a Superdex 200 10/300 GL column with an elution volume of around 16 ml, indicating that it has a molecular weight of approximately 50 kDa. SDS-PAGE showed that the molecular weight of the E165R protein monomer is 17 kDa (Fig. 1A). Sedimentation velocity analytical ultracentrifugation analyses further confirmed that the E165R protein exists as a homotrimer (∼50.5 kDa) in solution (Fig. 1B). In the enzyme activity assay, we found that E165R specifically hydrolyzed dUTP and produced pyrophosphate (PPi), but did not hydrolyze dATP, dTTP, dCTP, or dGTP (Fig. 1C). We further determined the value of Michaelis constant (*K_m_*), which was 3.788 ± 0.191 mΜ (Fig. 1D). These results suggested that the purified ASFV E165R protein is a homotrimeric dUTPase with strict substrate specificity.

### Crystal structure of ASFV E165R.

**FIG 2:**
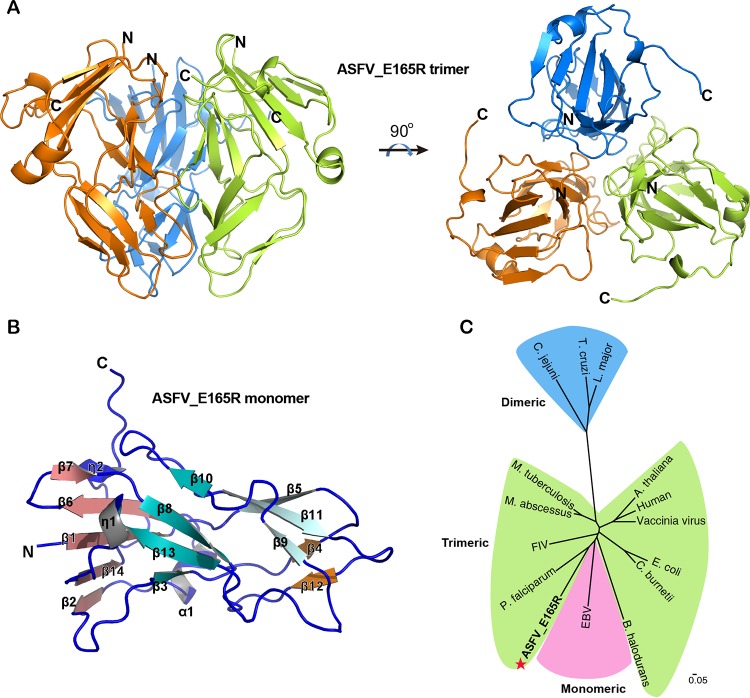
Crystal structure of apo-E165R. (A) Side and top views of the trimeric apo-E165R structure. The three protomers are colored in orange, green, and sky blue, respectively. (B) Structure of a protomer of apo-E165R. α, α-helix; β, β-strand; η, 3_10_ helix. The 14 β-strands form 4 β-sheets, which are indicated by salmon, teal, orange, and pale cyan. (C) Phylogenetic tree of dUTPases from different species. Phylogenetic assay shows that the protein sequences of dUTPases from different species cluster into three groups corresponding to the monomeric, dimeric, and trimeric dUTPase families. ASFV E165R (labeled with a red star) belongs to the trimeric dUTPase group.

The structure of ASFV E165R was solved by molecular replacement, using the structure of P. falciparum dUTPase as a search model, to a resolution of 2.30 Å, with *R*_work_ and *R*_free_ values of 22.2% and 25.5%, respectively (Table 1). The structure is a closely packed 3-fold symmetric homotrimer, with one E165R trimer in the asymmetric unit (Fig. 2A). There are 447 visible residues (residues 1 to 149 of the total 165 residues in each subunit) in this structure, which account for 90.3% of the intact protein, and 16 residues at the C terminus of each subunit are missing due to the poor electron density. The E165R protomer in the trimer is mainly composed of β-strands. The 14 β-strands (β1 to β14) form four sheets: β1, β2, β6, β7, and β14 form a parallel/antiparallel mixed sheet, β3, β8, β10, and β13 form an antiparallel sheet, β4 and β12 form another antiparallel sheet, and β5, β9 and β11 form the third antiparallel sheet. One α-helix (α1) and two 3_10_ helices (η1 and η2) appear after β1, β13, and β14, respectively (Fig. 2B; see also Fig. S1A).

**TABLE 1:** Statistics for crystallographic data collection and structure refinement

Parameter	Value	
	E165R	E165R/dUMP
Data collection		
Space group	I2_1_3	P43
Cell dimensions		
a, b, c (Å)	97.86, 97.86, 90.00	57.42, 57.42, 149.53
α, β, γ (°)	90.00, 90.00, 90.00	90.00, 90.00, 90.00
Resolution (Å)	50.00–2.30 (2.38–2.30)^*a*^	50.00–1.70 (1.76–1.70)^*a*^
*R*_pim_^*b*^	0.015 (0.013)	0.030 (0.026)
*CC*_1/2_	1.000 (0.999)	0.997 (0.997)
*I*/σ*I*	53.90 (7.33)	24.81 (3.07)
Completeness (%)	100.0 (100.0)	100.0 (100.0)
Redundancy	38.6 (38.7)	13.8 (14.0)
Refinement		
Resolution (Å)	39.95–2.30	37.64–1.70
No. of reflections	7,082	53,231
*R*_work_/*R*_free_^*c*^	0.222/0.255	0.188/0.215
No. of atoms		
Protein	3,471	12,038
Ligand/ion		60
Water	152	450
*B*-factors		
Protein	30.6	17.2
Ligand/ion		16.7
Water	30.4	27.0
RMSD		
Bond lengths (Å)	0.0	0.0
Bond angles (°)	0.6	0.7
Ramachandran analysis		
Favored (%)	95.21	99.06
Allowed (%)	4.79	0.70
Outliers (%)	0.00	0.23

a Values in parentheses are for highest-resolution pshells.

b $R_{\text{pim}}=\Sigma_{\text{hkl}}{\left[\text{1}/\left(\text{N}-\text{1}\right)\right]}^{\text{1}/\text{2}}\Sigma_{\text{i}}|I_{\text{i}}|/\Sigma_{\text{hkl}}\Sigma_{\text{i}}I_{\text{i}}$, where *I*_i_ is the observed intensity and is the average intensity from multiple measurements.

c $R_{\text{work}}=\Sigma ||F_{\text{o}}|-|F_{\text{c}}||/\Sigma |F_{\text{o}}|$, where *F*_o_ and *F*_c_ are the structure-factor amplitudes from the data and the model, respectively. *R*_free_ is the *R* factor for a subset (5%) of reflections that was selected prior to refinement calculations and was not included in the refinement.

10.1128/mBio.02483-19.1 FIG S1 Comparison between the apo-E165R and the E165R-dUMP complex structures. (A) Diagram of the secondary structures for apo-E165R. (B) Diagram of the secondary structures for E165R protein in the E165R-dUMP complex. (C) Alignment of one subunit from the apo-E165R (deepoliver) and the E165R-dUMP complex (deepteel) structures. The C termini of these two structures are in the red dashed circle. (D) Alignment of the apo-E165R (deepoliver) and the E165R-dUMP (deepteel) trimers. The angle degrees between the principal axes of the apo-E165R subunit (orange) and those of the E165R-dUMP subunit (cyan) are indicated. (E) Substrate binding pocket of the apo-E165R. The surface representation of the apo-E165R is shown and the binding pocket is colored orange. The area and volume of the pocket are indicated. The cartoon representations of aligned apo-E165R and the E165R-dUMP are shown as sky blue and cyan, respectively. The dUMP, its contact residues in the E165R-dUMP, and their corresponding counterparts in the apo-E165R are shown as sticks. (F) The substrate/product binding pocket of E165R-dUMP complex. The water molecules (oxygen atoms, red sphere; hydrogen atoms, white sphere) binding in the pocket and their polar contacts (yellow dashed line) with dUMP and the pocket residues are shown. The water molecule in the blue dashed circle directly forms a hydrogen bond with the phosphoric acid group of the dUMP and may be an active water molecule that is involved in hydrolysis reaction. Download FIG S1, TIF file, 2.1 MB. Copyright © 2019 Li et al. 2019 Li et al. This content is distributed under the terms of the Creative Commons Attribution 4.0 International license.

Phylogenetic analysis shows that primary sequences of dUTPases from different species are divided into three clusters, with each cluster corresponding to an oligomeric form (monomer, dimer, or trimer). ASFV E165R falls into the cluster of trimeric dUTPases (Fig. 2C). Therefore, combining the ultracentrifugation assay, the enzymatic assay, the crystal structure, and the phylogenetic analysis, we can conclude that ASFV E165R is a member of the trimeric dUTPase family.

### Crystal structure of the E165R-dUMP complex.

**FIG 3:**
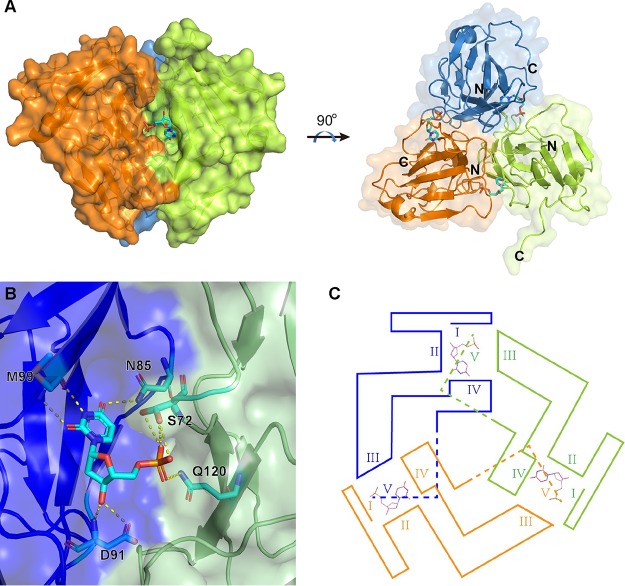
Crystal structure of E165R in complex with dUMP. (A) The overall view of the trimeric E165R-dUMP complex. The three protomers are colored according to the colors shown in Fig. 2A. dUMP substrates are shown as sticks in cyan. (B) Detailed view of one dUMP binding pocket. The binding pocket is formed by two protomers of E165R homotrimer, which are colored in blue and green. (C) The position relations of the motifs (I, II, III, and IV) that surround the substrate binding pockets of E165R.

To further define the active center and explore the catalyzing mechanism of E165R as a dUTPase, we made an attempt to determine the crystal structure of E165R in complex with the dUTP substrate. However, we obtained the crystal structure of E165R-dUMP complex rather than E165R-dUTP complex at a resolution of 1.70 Å, with *R*_work_ and *R*_free_ values of 18.8% and 21.5%, respectively (Table 1). This result further confirmed that E165R has a pyrophosphatase activity that degrades dUTP into dUMP and pyrophosphoric acid. In the complex, the E165R has an overall similar trimeric three-fold symmetric structure to that of apo-E165R. Each of the three dUMP binds to the interface of two protomers of the E165R trimer (Fig. 3A).

All three E165R subunits in the crystal structure of E165R-dUMP complex contain 1 to 144 residues of the total 165 residues of E165R, and the 21 residues at the C terminus are invisible due to poor electronic density. The secondary structure of the E165R protein in the E165R-dUMP complex is very similar to that of apo-E165R, except that the C-terminal 3_10_ helix in apo-E165R is missing in the E165R-dUMP complex (Fig. S1B).

By aligning one single subunit from apo-E165 and from the E165R-dUMP complex, we showed that these two subunit structures overlap well. Only their C termini show obvious conformational differences (Fig. S1C), suggesting high flexibility of the C terminus of E165R. By aligning the apo-E165R and the E165R-dUMP complex trimers, we found that their subunits do not precisely overlap. There are certain anticlockwise rotations of each E165R-dUMP subunit relative to their corresponding subunits in apo-E165R. The angle degrees between the principal axes of the corresponding subunits in apo-E165R and in the E165R-dUMP complex are 1.96°, 4.87°, and 4.97° (Fig. S1D).

dUMP interacts with N85, G88, L89, I90, D91, Y94, and M99 of one E165R protomer and R71, S72, and Q120 of another protomer. Among these interactions, N85, D91, and M99 from one protomer form hydrogen bonds with the deoxyuridine part of dUMP, while S72 and Q120 from the other protomer form hydrogen bonds with the phosphate group of dUMP (Fig. 3B).

dUTPases contain five conserved sequence motifs, which line the substrate binding pocket (active center) of the enzymes (25). By aligning E165R and other trimeric dUTPases, the five motifs in E165R were defined (see Fig. S2). As for E165R, motifs I, II, and IV of the active center are contributed by one protomer while motif III is contributed by another protomer. Like in many other dUTPase structures, the C termini (which contain motif V) of the three protomers were omitted due to poor electron density (Fig. 3C). By comparing the primary sequences of the five motifs from E165R and other trimeric dUTPases, we found that S72 from motif II, D91 from motif III, and Q120 from motif IV, which are responsible for forming hydrogen bonds with dUMP, are highly conserved among dUTPases from different species (Fig. S2).

**FIG 4:**
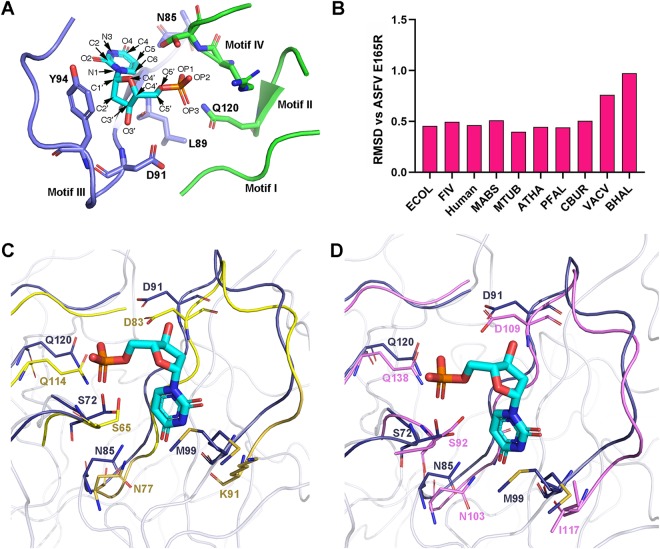
Comparison of the structures of active center motifs of different trimeric dUTPases. (A) The four enzyme active center motifs (I, II, III, and IV) of E165R. The atom names of the dUMP are indicated and side chains of the residues that form atom contacts with the dUMP are shown as sticks. (B). The RMSD values between motifs I, II, III, and IV from ASFV E165R and from other trimeric dUTPases. (C) Superimposition of the active center motifs (I, II, III, and IV) from ASFV E165R (blue) with those of M. tuberculosis dUTPase (yellow). The side chains of the residues that form hydrogen bonds with dUMP in ASFV E165R (please refer to Fig. S1B in the supplemental material) and those of their corresponding aligned residues in M. tuberculosis are shown as sticks. (D) Superimposition of the active center motifs (I, II, III, and IV) from ASFV E165R (blue) with those of P. falciparum dUTPase (pink). The side chains of the residues that form hydrogen bonds with dUMP in ASFV E165R (please refer to Fig. 2) and those of their corresponding aligned residues in P. falciparum are shown as sticks. ECOL, Escherichia coli; MABS, Mycobacterium abscessus; MTUB, Mycobacterium tuberculosis; ATHA, Arabidopsis thaliana; PFAL: Plasmodium falciparum; CBUR, Coxiella burnetii; VACV, *Vaccinia virus*; BHAL, Bacillus halodurans.

10.1128/mBio.02483-19.2 FIG S2 Sequence alignment of dUTPases in the trimeric dUTPase family. FIV, feline immunodeficiency virus; HSAP, Homo sapiens; ECOL, Escherichia coli; MABS, Mycobacterium abscessus; MTUB, Mycobacterium tuberculosis; ATHA, Arabidopsis thaliana; PFAL, Plasmodium falciparum; BHAL, Bacillus halodurans; CBUR, Coxiella burnetii; VACV, *Vaccinia virus*. The five conserved motifs are indicated. The red boxes show the positions where ASFV E165R forms hydrogen bonds with its ligand (please refer to Fig. 4). Download FIG S2, TIF file, 1.3 MB. Copyright © 2019 Li et al. 2019 Li et al. This content is distributed under the terms of the Creative Commons Attribution 4.0 International license.

By comparing the substrate/product binding pockets of apo-E165R and the E165R complex, we found that the binding pocket of apo-E165R is much larger than that of the E165R complex. As calculated by CASTp (26), the solvent accessible area and volume of the binding pockets in apo-E165R are 249.3 Å^2^ and 163.2 Å^3^, respectively (Fig. S1E). In contrast, these values become 127.0 Å^2^ and 62.2 Å^3^, respectively, for the binding pockets in the E165R-dUMP complex (Fig. S1F). Obviously, the binding pocket of E165R remarkably shrinks after binding the substrate/product for better coordinating them. There are several water molecules in the ligand binding pockets of both apo-E165R and the E165R complex structures. Some water molecules in the ligand binding pocket of the E165R-dUMP complex form hydrogen bonds with the phosphoric acid group of dUMP. They may be the active water molecules that are involved in the hydrolysis mechanism (Fig. S1F). Magnesium ions are important for the catalysis mechanism by coordinating the hydrolysis intermediate conformation and maximizing the catalyzing efficiency (25). However, we did not found any magnesium ion, free pyrophosphoric, or phosphoric acid groups in the binding pocket of the E165R-dUMP complex, just like in other dUTPase-dUMP complexes (11). The magnesium ion and the PP_i_ (one of the hydrolysis products) may have been released from the active center after the hydrolysis reaction finished.

### Comparison of the structure of E165R and other trimeric dUTPases.

In the E165R-dUMP complex, the dUMP is mainly coordinated by the active center motifs I, II, III, and IV (Fig. 4A). Based on the root mean square deviation (RMSD) values between the active center motifs I, II, III, and IV of E165R and those of other trimeric dUTPases, we found that the configuration of the active center of E165R is most similar to those of M. tuberculosis and P. falciparum dUTPases (Fig. 4B).

By superimposing the active center motifs of ASFV E165R with those of M. tuberculosis, we can see that the conformations of the backbones of the four motifs constituting the active centers of E165R and the M. tuberculosis dUTPase are very similar. In addition, the side chains of key residues that interact with the substrate in these motifs have the same orientations with those of M. tuberculosis dUTPase. Specifically, the side chains of S72, N85, D91, M99, and Q120 in E165R, which form hydrogen bonds with dUMP, have the same orientations as those of S65, N77, D83, K91, and Q114 in the M. tuberculosis dUTPase. These results further demonstrate the similarity of the active centers of E165R and M. tuberculosis dUTPase (Fig. 4C).

Similarly, alignment of the four active center motifs (motifs I, II, III, and IV) of ASFV E165R with those of the P. falciparum dUTPase shows that conformations of the backbones of these active center motifs as well as the side chain orientations of the substrate-interacting residues in these motifs of E165R are also very similar to those of P. falciparum dUTPase (Fig. 4D).

**FIG 5:**
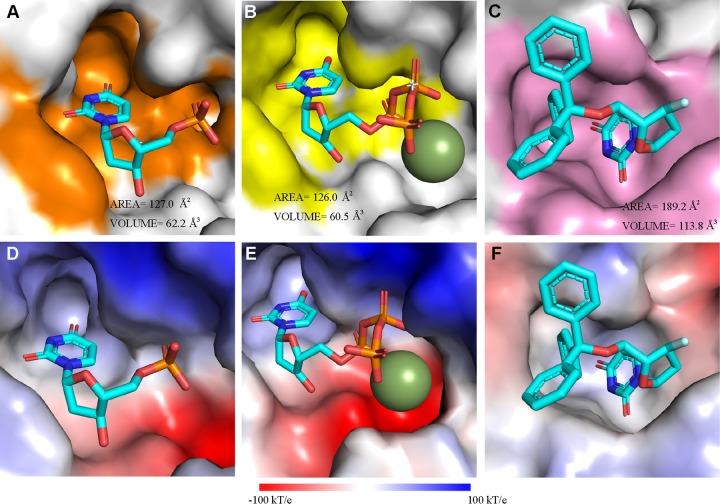
Comparison of the E165R structure with those of other trimeric dUTPases in complex with substrates or inhibitors. The substrate binding pockets in E165R in complex with dUMP (A), M. tuberculosis dUTPase in complex with dUTP (PDB ID 1SM8) (B), and P. falciparum dUTPase in complex with dUPNPP (PDB ID 1VYQ) (C) are shown. The pocket-forming residues in panels A, B, and C are colored orange, yellow, and pink, respectively. The solvent accessible areas and volumes of the pockets calculated by CASTp (27) are indicated. The electrostatic potential distribution mapped to the solvent-accessible surface of E165R (D), M. tuberculosis dUTPase (E), and P. falciparum dUTPase (F) are shown. The color gradient bar for the electrostatic surface potential in panels D, E, and F is shown from red (acidic) to blue (basic) at the bottom (k, the Boltzmann constant; T, temperature; e, the charge of an electron).

We also calculated the solvent accessible surface areas and volumes of the substrate binding pockets of E165R in complex with dUMP, M. tuberculosis dUTPase in complex with dUTP, and P. falciparum dUTPase in complex with α,β-imido-dUTP (dUPNPP) by CASTp (26), and the results show that areas and volumes of E165R and M. tuberculosis dUTPase substrate binding pockets are very similar and that the shapes of the substrate binding pockets of these two proteins are also very similar (Fig. 5A and B). However, the shape, area, and volume of the binding pocket of dUPNPP in P. falciparum dUTPase show obvious differences from those of E165R and M. tuberculosis dUTPase (Fig. 5C). In addition, we found the electrostatic potentials are distinct among these three pockets (Fig. 5D to F). These observations suggest that the unique property of the substrate binding pocket should be considered in the development of inhibitors against ASFV E165R.

## DISCUSSION

Emerging and reemerging viruses keep challenging public health (27). ASFV is highly contagious and causes lethal diseases in both domestic pig and wild boar victims. Identification of drug targets from ASFV and structure-based drug design are important strategies for ASFV control and prevention. As a dUTPase, E165R plays an essential role in maintaining viral genome fidelity and integrity. Depletion of E165R aborts productive replication of ASFV in swine macrophages (28). Due to the high level of dUTP or a high ratio of dUTP/dTTP in macrophages, sufficient dUTPase activity is critical for viruses to replicate successfully in macrophages (29, 30). In addition to strictly regulating the dUTP level and keeping uracil out of DNA, dUTPases from some virus species have another function as a signaling protein. Epstein-Barr virus (EBV) dUTPase binds to cellular Toll-like receptor 2 and activates NF-κB expression, which leads to immune dysregulation in the host (31). Murine herpesvirus 68 (MHV-68) dUTPase is essentially involved in the viral infective process by blocking the type I interferon signaling pathway via a mechanism independent of its intrinsic dUTPase activity. Whether ASFV E165R has similar functions needs to be explored in the future.

Our biochemical and crystallographic results demonstrate that ASFV E165R is a trimeric dUTPase with pyrophosphatase activity. The product of the hydrolysis catalyzed by E165R, dUMP, binds in the active center composed of motifs from the two adjacent protomers. The high-resolution structure of E165R-dUMP gives in-depth architecture information of the E165R active center and therefore provides a unique opportunity for designing inhibitors of this enzyme. By comparing the active center of E165R with those of other trimeric dUTPases, we found that both the architecture and substrate binding geometry of the E165R active center are very similar to those of M. tuberculosis and P. falciparum dUTPases, despite the binding pockets having distinct electrostatic potentials. Some chemicals have been designed to inhibit the M. tuberculosis dUTPase, such as α,β-imido-dUTP (dUPNPP) (32). P. falciparum dUTPase has also been chosen as a target for antimalarial drug design, and a series of triphenylmethane derivatives of deoxyuridine were found to have antimalarial activities (21). These chemicals may also be effective for inhibiting E165R dUTPase activity or could serve as lead compounds for designing novel anti-ASFV drugs. All these observations need further investigation in the future.

## MATERIALS AND METHODS

### Gene cloning, protein production, and purification.

The ASFV E165R gene (GenBank accession number CBW46796.1), coding for the dUTPase, fused at its C terminus with a hexa-histidine tag was cloned into the pET-21a vector (Novagen) with NdeI and XhoI restriction sites (33). Transformed E. coli strain BL21(DE3) clones were grown in LB medium containing 100 μg/ml ampicillin to an optical density at 600 nm (OD_600_) of 0.6 to 0.8 at 37°C. Expression of the recombinant proteins was induced by the addition of 0.5 mM isopropyl-β-d-1-thiogalactopyranoside (IPTG), and incubation was continued for a further 16 h at 16°C. Cells were harvested by centrifugation at 7,000 × *g* for 15 min at 4°C, resuspended in lysis buffer (20 mM Tris-HCl [pH 8.5] and 150 mM NaCl), and further homogenized with a low-temperature ultrahigh pressure cell disrupter (JNBIO, China). The lysate was clarified by centrifugation at 20,000 × *g* for 60 min at 4°C. The supernatant was purified by metal affinity chromatography using a HisTrap HP 5 ml column (GE Healthcare). Proteins were eluted using the lysis buffer supplemented with 300 mM imidazole. The proteins were further purified by gel filtration chromatography using a HiLoad 16/60 Superdex 75 PG (GE Healthcare) with a running buffer of 20 mM Tris-HCl (pH 8.5) and 50 mM NaCl, and the collected protein fractions were concentrated to 10 mg/ml using a membrane concentrator with a molecular weight cutoff of 10 kDa (Millipore).

### Crystallization, data collection, and structure determination.

Crystallization trials were set up with commercial crystallization kits (Hampton Research) using the sitting drop vapor diffusion method. Typically, 1 μl protein was mixed with 1 μl reservoir solution. The resultant drop was then sealed, equilibrating against 100 μl reservoir solution at 18°C. Diffractable crystals of ASFV E165R were obtained in 0.1 M ammonium citrate tribasic (pH 7.0), 12% (wt/vol) polyethylene glycol 3350 at 18°C. For the ASFV E165R-dUMP complex, 10 mg/ml E165R proteins were cocrystallized with 10 mM dUTP and 10 mM MgCl_2_ in a buffer of 20 mM Tris-HCl (pH 8.5) and 50 mM NaCl at 18°C. Diffractable crystals of the ASFV E165R-dUMP complex were obtained in 0.1 M citric acid (pH 3.5), 14% (wt/vol) polyethylene glycol 1000. Crystals were flash-cooled in liquid nitrogen after a brief soaking in reservoir solution with the addition of 17% (vol/vol) glycerol. The X-ray diffraction data were collected under cryogenic conditions (100 K) at Shanghai Synchrotron Radiation Facility (SSRF), beamline BL19U1, and indexed, integrated, and scaled with HKL2000 (34).

The E165R and E165R-dUMP complex structures were solved by the molecular replacement method using Phaser (35) from the CCP4 program suite (36), with the structure of P. falciparum dUTPase in complex with 2,3-deoxy-3-fluoro-5-*o*-trityluridine (PDB 1VYQ) as the search model (21). Initial restrained refinement and manual model building were performed using REFMAC5 (37) and COOT (38), respectively. Further refinement was performed using Phenix (39). Final statistics for data collection and structure refinement are represented in Table 1.

### Biochemical characterization of E165R protein.

The purified protein was analyzed with an analytical gel-filtration assay with a calibrated Superdex 200 10/300 GL column (GE Healthcare). The sample was further analyzed with SDS-PAGE.

The analytical ultracentrifugation assay was performed according to a previously reported method (40). The proteins were prepared in 20 mM Tris (pH 8.5) and 150 mM NaCl at an *A*_280_ of 0.8. The assay was performed on an optimal ProteomeLab XL-I analytical ultracentrifuge (Beckman Coulter) at a speed of 48,000 rpm. The molecular mass analysis was performed with the XL-I data analysis software.

### Measurement of enzyme kinetics.

The substrate specificity of dUTPase ASFV E165R was determined by monitoring the production of PP_i_ according to a previously reported method (41). The dUTPase enzymatic activity assay was performed by monitoring the color change of cresol red using a SpectraMax M5 (Molecular Devices, USA) (42). Briefly, a total of 990 μl of buffer solution (2 mM bicine, 100 mM KCl, 5 mM MgCl_2_, pH 8.0) including final concentrations of 25 μM cresol red and 10 μM dUTP was rapidly mixed with 10 μl of 2.5 μM dUTPase in a cuvette (optical path of 1 cm). The color change of cresol red was recorded by monitoring the absorbance at 573 nm at intervals of 1.0 s at 37°C. Finally, the *K_m_* values were calculated using the integrated Michaelis-Menten method.

### Data availability.

The accession numbers for the atomic coordinates and diffraction data reported in this paper are PDB 6KY8 (crystal structure of E165R) and 6KY9 (crystal structure of E165R/dUMP complex).
