# Supplementary figures and images for: Crystal Structure of African Swine Fever Virus dUTPase Reveals a Potential Drug Target
Source: mBio. 2019 Oct 29;10(5):e02483-19. doi: 10.1128/mBio.02483-19 (PMC6819664; doi:10.1128/mBio.02483-19)

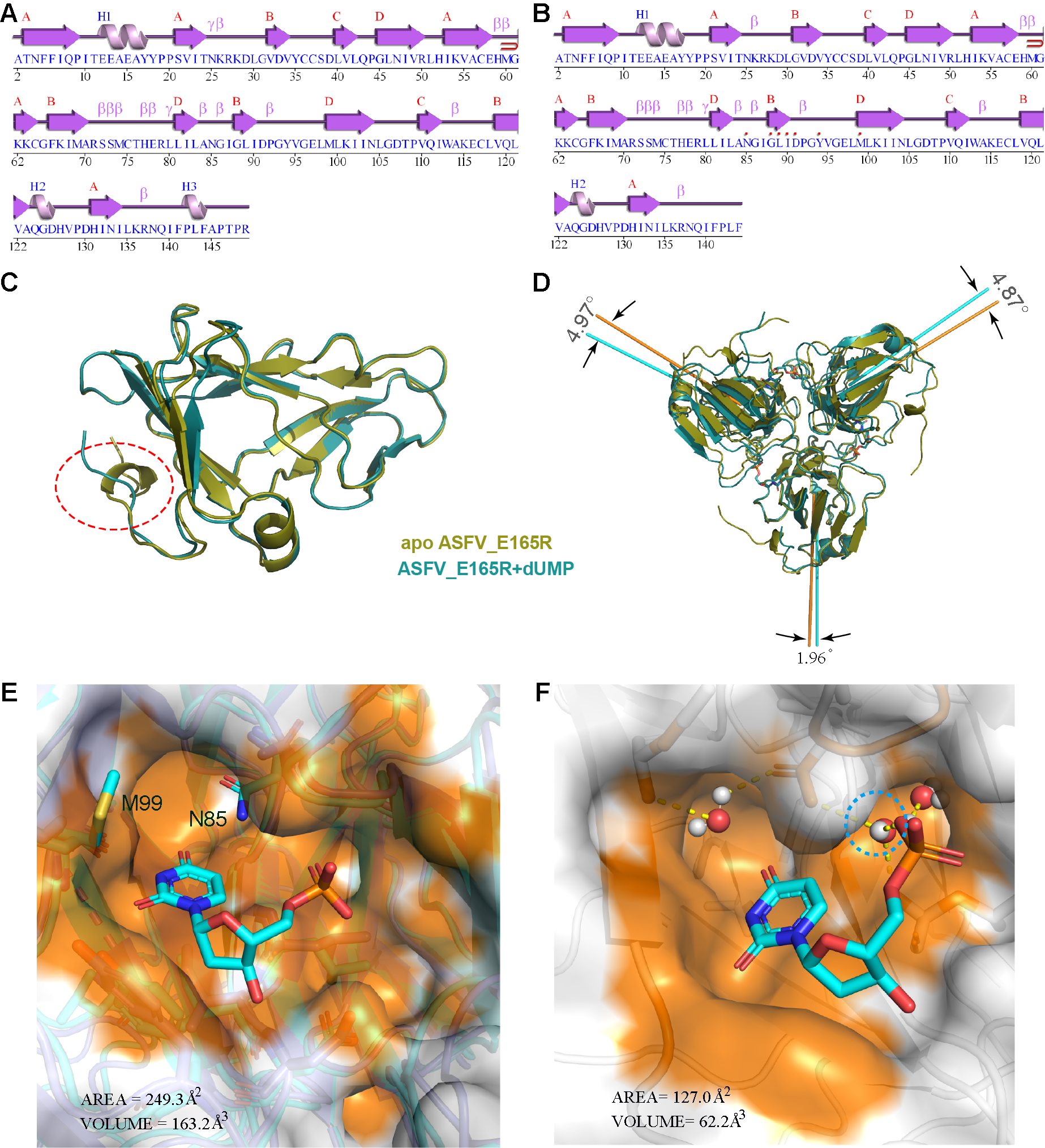

Supplement: FIG S1 [file mBio.02483-19-sf001.tif]

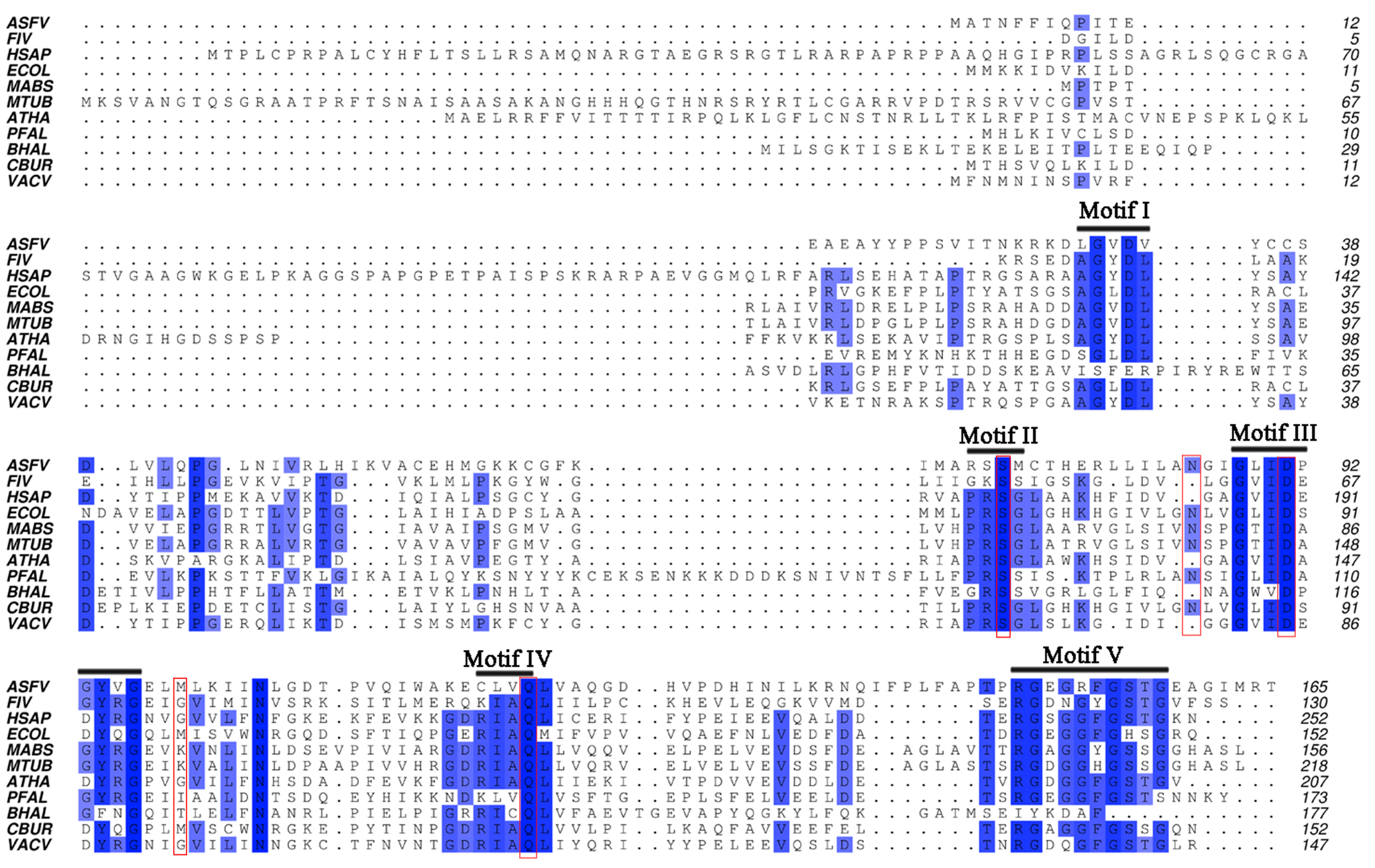

Supplement: FIG S2 [file mBio.02483-19-sf002.tif]
